# Identification and exploration of novel M2 macrophage-related biomarkers in the development of acute myocardial infarction

**DOI:** 10.3389/fcvm.2022.974353

**Published:** 2022-11-10

**Authors:** Qiaoyu Zhou, Guogang Zhang, Zhaoya Liu, Jiayi Zhang, Ruizheng Shi

**Affiliations:** ^1^Department of Cardiovascular Medicine, The Third Xiangya Hospital of Central South University, Changsha, China; ^2^Department of Geriatrics, The Third Xiangya Hospital, Central South University, Changsha, China; ^3^Department of Gastroenterology, The First Hospital of Changsha, Changsha, China; ^4^Department of Cardiovascular Medicine, The Xiangya Hospital, Central South University, Changsha, China

**Keywords:** acute myocardial infarction, CIBERSORT, weighted gene co-expression network analysis, M2 macrophage, diagnostic biomarker genes, bioinformatics

## Abstract

**Background:**

Acute myocardial infarction (AMI), one of the most severe and fatal cardiovascular diseases, is a major cause of morbidity and mortality worldwide. Macrophages play a critical role in ventricular remodeling after AMI. The regulatory mechanisms of the AMI progression remain unclear. This study aimed to identify hub regulators of macrophage-related modules and provide translational experiments with potential therapeutic targets.

**Materials and methods:**

The GSE59867 dataset was downloaded from the Gene Expression Omnibus (GEO) database for bioinformatics analysis. The expression patterns of 22 types of immune cells were determined using CIBERSORT. GEO2R was used to identify differentially expressed genes (DEGs) through the limma package. Then, DEGs were clustered into different modules, and relationships between modules and macrophage types were analyzed using weighted gene correlation network analysis (WGCNA). Further functional enrichment analysis was performed using significantly associated modules. The module most significantly associated with M2 macrophages (Mϕ2) was chosen for subsequent analysis. Co-expressed DEGs of AMI were identified in the GSE123342 and GSE97320 datasets and module candidate hub genes. Additionally, hub gene identification was performed in GSE62646 dataset and clinical samples.

**Results:**

A total of 8,760 DEGs were identified and clustered into ten modules using WGCNA analysis. The blue and turquoise modules were significantly related to Mϕ2, and 482 hub genes were discerned from two hub modules that conformed to module membership values > 0.8 and gene significance values > 0.25. Subsequent analysis using a Venn diagram assessed 631 DEGs in GSE123342, 1457 DEGs in GSE97320, and module candidate hub genes for their relationship with Mϕ2 in the progression of AMI. Finally, four hub genes (CSF2RB, colony stimulating factor 2 receptor subunit beta; SIGLEC9, sialic acid-binding immunoglobulin-like lectin 9; LRRC25, leucine-rich repeat containing 25; and CSF3R, colony-stimulating factor-3 receptor) were validated to be differentially expressed and to have high diagnostic value in both GSE62646 and clinical samples.

**Conclusion:**

Using comprehensive bioinformatics analysis, we identified four novel genes that may play crucial roles in the pathophysiological mechanism of AMI. This study provides novel insights into the impact of macrophages on the progression of AMI and directions for Mϕ2-targeted molecular therapies for AMI.

## Introduction

Acute myocardial infarction (AMI) is one of the most severe manifestations of cardiovascular disease, affecting an estimated 7.29 million people in 2015 and remarkably aggravating the global health burden (1, 2). However, the causes and pathophysiology of AMI remain unclear. The greater the life expectancy of the population, the more serious the need for research aimed at studying the underlying molecular mechanisms of AMI. Likewise, we should identify novel diagnostic biomarkers and therapeutic targets to improve the early diagnosis and treatment of AMI to improve the survival rates and quality of life in patients. Numerous studies have shown that immune cells play a crucial role in the symptomatology and pathophysiology of cardiovascular disease (3). Additionally, recent research has established that neutrophils amplify granulopoiesis in myocardial infarction (4), which suggests that recognizing changes in the peripheral blood during AMI may provide a novel therapeutic approach for AMI.

Similarly, the polarization/distribution of pro-inflammatory M1 macrophages (Mϕ1) and anti-inflammatory/reparative M2 macrophages (Mϕ2) also plays a significant role in the development of AMI (5). The microenvironment of AMI can influence the polarization state of macrophages (Mϕs), with a significant increase in Mϕ1 and a significant reduction in Mϕ2 in the beginning of AMI (5). An abnormal increase and activation of Mϕ2 can inhibit the aberrant gene expression associated with the myocardial remodeling (6). By secreting cytokines [e.g., interleukin (IL)-10, and transforming growth factor-beta (TGF-β) family members], Mϕ2 inhibits inflammation and activates fibroblasts to affect the balance between matrix metalloproteinases (MMPs) and tissue inhibitors of metalloproteinases (TIMPs) (7). Because of the insufficient regenerative capacity of the myocardium, this process is crucial for preventing the rupture or overdistention of the fragile and infarctional ventricular wall. Overall, the composition of Mϕ in heart tissue is heterogeneous in homeostasis and highly dynamic after injury (8). Therefore, identifying potential Mϕ2 associated biomarkers can not only help to establish their role in the immune system during the progression of AMI, but also contribute to the management and treatment of AMI patients.

In recent years, with the development and accessibility of various online comprehensive bioinformatics tools, the identification of distinct molecular markers and signaling pathways for different diseases has become easier (9, 10). Weighted gene co-expression network analysis (WGCNA), one of the most valuable and extensively used tools, has been used to establish a robust gene co-expression network and to identify hub gene modules that drive key cellular signaling pathways for diseases (11). Liu et al. identified specialized ferroptosis and hypoxia-associated co-expression networks for AMI, and nine hub genes were identified as potential prognostic biomarkers using WGCNA (12). Niu et al. utilized WGCNA to identify six co-expression modules in AMI and found that the *BCL3, PPIF, S100A9, HCK, PPIF, TBC1D9B*, and *SERPINA1* genes were hub genes for heart failure development (13). Qi et al. analyzed WGCNA to yield eight co-regulated gene clusters in coronary artery disease (CAD), and three genes closely related to CAD showed potential molecular mechanisms (14). However, no robust Mϕ-related gene co-expression network has been established during AMI progression.

In this study, we chose the GSE59867 dataset, which contains stable coronary artery disease (SCAD) and AMI-related gene expression data downloaded from the Gene Expression Omnibus (GEO) database, to identify potential Mϕ-related biomarkers of AMI using WGCNA analysis. Similarly, CIBERSORT, which has been widely applied to estimate the infiltration of immune cells in various diseases, was used to analyze different types of Mϕ in AMI, identify the most significant modules related to Mϕ infiltration, and further analyze and verify the diagnostic genes of these modules. Using the WGCNA and CIBERSORT analyses, we aimed to construct a gene co-expression network of the Mϕ traits and identify hub genes involved in the progression of AMI.

## Materials and methods

### Data sources

**FIGURE 1:**
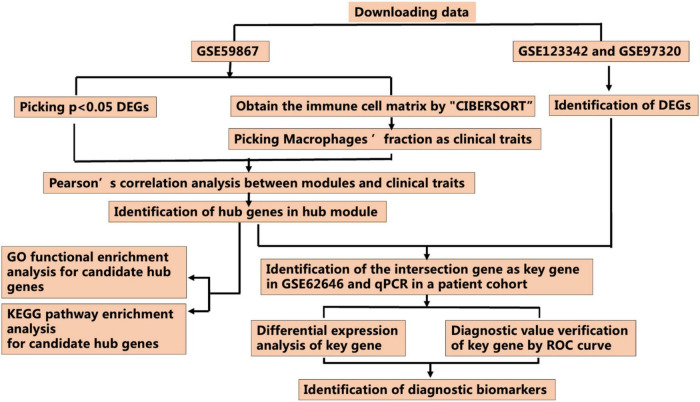
Workflow for identification of the novel M2 macrophage-related biomarkers through the CIBERSORT and weighted gene correlation network analysis (WGCNA) algorithms (DEGs, differentially expressed genes; ROC curve, receiver operating characteristic curve).

Figure 1 presents the workflow of this study. The GSE59867, GSE123342, GSE97320, and GSE62646 datasets were obtained from the GEO database^1^. The GSE59867 dataset, obtained at admission after AMI and containing the transcriptional profile of peripheral blood mononuclear cells (PBMCs), was selected for further analysis. The GPL6244 platform was used for data sequencing [Affymetrix Human Gene 1.0 ST Array, transcript (gene) version]. The GSE123342 dataset, including 65 patients with AMI and 22 patients with stable CAD, was created using the GPL17586 platform [Affymetrix Human Transcriptome Array 2.0, transcript (gene) version]. The GSE97320 dataset, which included three patients with AMI and three healthy individuals, was generated using the GPL570 platform [Affymetrix U133A microarray]. The GSE62646 dataset, which included 28 patients with myocardial infarction on admission and 14 patients with SCAD, was performed using the GPL6244 platform [Affymetrix Human Gene 1.0 ST Array transcript (gene) version]. CIBERSORT, which can estimate the relative expression of 22 immune cell types (including three phenotypes of Mϕs) as a bioinformatics algorithm, was used to evaluate Mϕ composition based on the gene expression matrix (15).

### Data pre-processing

GEO2R, as a tool provided by the GEO database depending on the limma package in R, was used to identify differentially expressed genes (DEGs) in each dataset. Adjusted *p*-values < 0.05 were used as cut-off criteria for screening out all DEGs in GSE59867, and further analysis of 8,760 DEGs was performed using WGCNA. Overexpressed DEGs in GES123342 and GES97320 were screened out, based on the cut-off criteria of |log2FC| > 0.5 and adjusted *p*-values < 0.05.

### Construction of weighted gene correlation network analysis

The WGCNA R package was used to perform the weighted correlation network analysis (11). First, the similarity of co-expression between genes *m* and *n* was defined as S_mn_ = |cor(m, n)|. The correlated adjacency of the genes was analyzed using the power function: a_mn_ = power (S_mn_, β) = |S_mn_| ^β^. A gradient method was used to test the mean connectivity and scale independence (the power values ranging from 1 to 20). A scale-free network was obtained from an appropriate power value, which was screened out by a degree of independence above 0.80 (11). Finally, a topological overlap matrix was transformed from the adjacency matrix, and analysis of hierarchical average linkage clustering was used to detect the modules in the gene dendrogram. In addition, we extracted the genes that were most closely related to each module for further analysis.

### Selection of key modules corresponding to macrophage infiltration levels

After identification of the modules, we further summarized the module eigengene (ME) using the module expression levels of the first principal component and estimated the relationships of module–Mϕ infiltration levels. The key module of the network was identified using two methods. In the first method, we calculated the Pearson correlation coefficients, which indicated the correlation between the MEs of each module and the relative expression of macrophages identified by the CIBERSORT, to permit the identification of modules that were most significantly related to the infiltration levels of Mϕ (*p* < 0.05), which we identified as Mϕ features. In the second method, we calculated the mean absolute Pearson correlation coefficients for all genes in the module, which indicated the gene significance (GS) between the expression levels of each gene and Mϕ features (11). The correlation between the module and the Mϕ features was enhanced by an increase in the mean absolute value. In the WGCNA, we selected the module with the highest correlation coefficient as the key module for further analysis (11).

### Identification of candidate hub genes in the key module

Hub genes, which are highly related to the nodes of a module, have crucial functions. We measured the interconnections between the genes and modules to screen hub genes through the module membership (MM) determination (11). For each gene, MM was defined as the correlation between the profile of gene expression profile and ME of a given module. For instance, the interconnection between hub gene i and the ME of the blue module was measured as MMblue(i) = cor(xi, Eblue), the higher the absolute value of MMblue(i), the greater was the connection between gene i and the blue module. Intramodular connectivity was highly correlated with MM measurements (11). In this study, we screened out candidate hub genes through the network screening function based on GS > 0.25 and MM > 0.8 in the WGCNA package.

### Functional enrichment analysis of hub modules

Gene Ontology (GO) function and Kyoto Encyclopedia of Genes and Genomes (KEGG) pathway enrichment analyses were performed using the clusterProfiler R package (version: 4.1.3) to determine the biological functions and signaling pathways of hub genes (16). The default parameters of the clusterProfiler R package were used, and *p* < 0.05 was set as the threshold for the recognition of the GO annotation and KEGG pathways of hub genes.

### Identification and recognition of real hub genes in other datasets

We extracted the independent GSE123342 and GSE97320 datasets (see text footnote 1), and subsequent analysis identified 12 overlapping genes between DEGs (|log2FC| > 0.5 and adjusted *p*-values < 0.05) in GSE123342 and GSE97320 and candidate genes in hub modules using a Venn diagram (Figure 7) as candidates for further analysis and validation. Additionally, GSE62646 was used to verify the messenger RNA (mRNA) expression of the hub genes. The GSE62646 dataset contained 98 blood samples, including 28 patients with AMI and 14 patients with SCAD. Based on gene expression levels, we calculated the area under the curve (AUC) of the receiver operating characteristic (ROC); the AUC was estimated using Wilcoxon-Mann–Whitney statistic, which is the simplest non-parametric estimator for constructing AUC confidence intervals (17). The real Mϕ2-related hub genes were defined as those with an AUC ≥ 0.80 (*p* < 0.05) in the ROC curve analysis, which was regarded as effective for distinguishing AMI and SCAD with remarkable specificity and sensitivity. Normality tests were performed using the Shapiro-Wilk and Kolmogorov−Smirnov tests. Normally distributed data were analyzed using the *t*-test; otherwise, the evaluation of DEGs between AMI and SCAD was performed using the Mann−Whitney U test.

### Verification of the clinical related genes by real-time reverse transcription PCR

All the protocols and the use of human bloods were in accordance with the Declaration of Helsinki and were approved by the Xiangya Hospital of Central South University Institutional Review Board. Five patients diagnosed with AMI and five healthy controls were enrolled in this study. The detailed characteristics of the patients are listed in Supplementary Table 1. Peripheral blood mononuclear cells were obtained from the AMI patients and healthy controls *via* density gradient centrifugation using the Ficoll reagents from Cytiva (America). According to manufacturer’s instruction, RNA extraction was performed using RNAex Pro reagent (Accurate, Changsha, China) and reverse transcription was performed using the Evo M-MLV RT Mix Kit (Accurate, Changsha, China), then, real-time quantitative PCR (qPCR) was carried out using SYBR^®^ Green Premix Pro Taq HS kit (Accurate, Changsha, China) on the ABI QuantStudio™ 5 real-time PCR system. The primer sequences are described in Supplementary Table 2. Normality tests were performed using the Shapiro-Wilk and Kolmogorov−Smirnov tests. Normally distributed data were analyzed using the *t*-test; otherwise, the evaluation of DEGs between AMI and controls was performed using the Mann−Whitney U test.

## Results

### Data processing strategy and mRNA expression profiles

The study protocol is illustrated in Figure 1. The mRNA expression profiles of AMI and SCAD patients or healthy controls in the GSE59867, GSE123342, GSE97320, and GSE62646 datasets were obtained from the GEO database, which was used to analyze DEGs separately in the following analysis.

**FIGURE 2:**
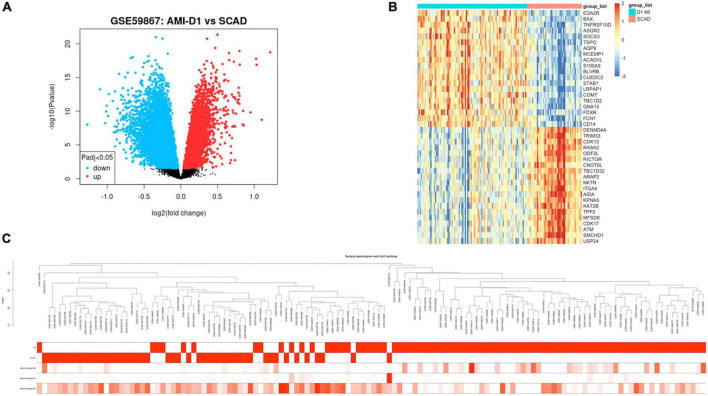
Expression profile of differentially expressed genes (DEGs). **(A)** Volcano map of gene expression levels. **(B)** Heatmap of top 20 up-regulated or down-regulated DEGs based on *p*-value. **(C)** Clinical feature heatmap and hierarchical clustering dendrogram of AMI or SCAD samples. The clinical characteristics heatmap represented by degree from dark red to white recognizes high to low levels of clinical characteristics, while gray indicates unavailable data.

As shown in Supplementary Table 3, 8,760 DEGs were identified between patients with AMI and SCAD, according to an adjusted *p*-value < 0.05. Among these DEGs in AMI, 4,607 genes were up-regulated, while another 4,153 genes were down-regulated. A volcano map of DEGs is shown in Figure 2A. The heatmap of the top 20 up-regulated DEGs and top 20 down-regulated DEGs were displayed in Figure 2B, and the specific DEGs are shown in Supplementary Table 4.

### Estimation of the Mϕ-infiltration level in acute myocardial infarction

The abundance of the Mϕ infiltration levels in all samples was analyzed by the CIBERSORT algorithm based on the gene expression matrix. Data of Mϕ features for WGCNA are composed of the clinical diagnosis and composition of the three Mϕ phenotypes (Supplementary Table 5).

### Construction of the co-expression network and identification of hub modules related to macrophages using weighted gene correlation network analysis

**FIGURE 3:**
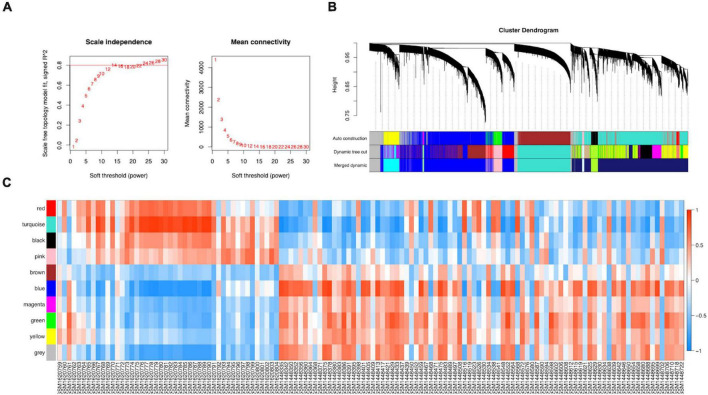
Construction of the co-expression network. **(A)** A scale-free co-expression network estimated by the soft-thresholding powers. **(B)** Ten co-expression modules constructed by clustering dendrograms of all DEGs based on topological overlap and assigned in different module colors (non-clustering DEGs shown in gray). **(C)** The relationships between modules and clinical samples.

The WGCNA R package was used to construct a gene co-expression network analysis of the 8,760 identified DEGs. We established a mean connectivity network and scale-independent topological network, with a soft-thresholding power of 14 and scale-free R2 of 0.80 (Figure 3A). A hierarchical clustering tree was constructed by splitting the dendrogram at correlative transition points using dynamic hybrid cutting. The leaves of the trees represented single genes, and branches of the dendrogram tree represented multiple genes with analogous expression. Branches were gene modules that included analogously expressed genes.

**FIGURE 4:**
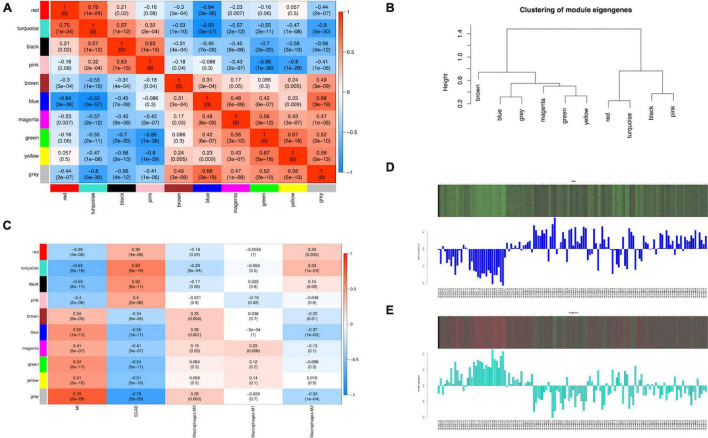
Consensus eigengene networks and their differential analysis. **(A)** Heatmap of the eigengene networks, red indicates positive correlation, and blue indicates negative correlation, the absolute value means the degree of adjacency with other modules. **(B)** The clustering trees (also called the dendrograms) of the consensus module eigengenes; **(C)** Relationship between consensus module and feature. Correlation analyses were conducted between clinical features and eigengene values represented for each module. The numbers in the matrix cell indicate the correlation coefficient and the related *p*-value. The blue module was most negatively related to Mϕ2, and the turquoise module was most positively correlated to Mϕ2. **(D,E)** The expression level of all samples in genes of blue **(D)** and turquoise **(E)** modules. In the heatmap, green indicates the low expression for samples, whereas red indicates the high expression, which means ME is strongly related with expression of genes in modules.

ME, the first principal component of each module, was a single value representing the highest percentage of expression values among all genes. After obtaining the MEs of all modules, we calculated the average distance and Pearson correlation coefficient between the MEs of all modules. The greater interconnection between modules was reflected by the greater relationship between the MEs representing the modules. Because of the average distance, average-linkage hierarchical clustering was used to perform cluster analysis on the recognized modules, and similar modules with a >0.75 correlation coefficient were merged to obtain 10 modules finally (Figure 3B). As shown in Supplementary Table 6, there were 2,482, 2,642, 1,441, 345, 187, 45, 83, 211, and 443 genes in the blue, turquoise, brown, green, black, magenta, pink, red, and yellow modules, respectively. The gray module included genes that were not clustered into a module, which was removed from the subsequent analysis. Figure 3C shows the relationships between the modules and samples. Figures 4A,B show the relationships and cluster trees of the modules, respectively.

**FIGURE 5:**
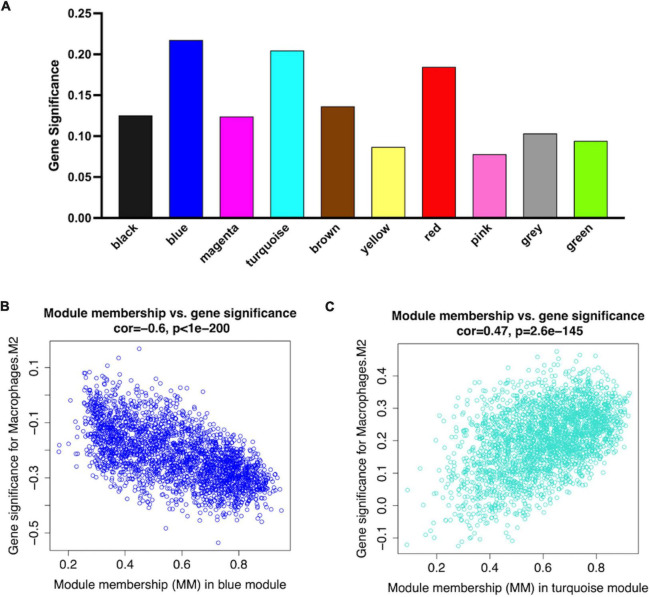
Network plots of module and eigengene. **(A)** Bar plots of mean GS in distinct modules. The higher value of mean GS indicates more significant correlation between macrophages M2 and the module **(B,C)**. Scatterplots in two hub modules with GS (*y*-axis) and MM (*x*-axis) separately.

The analysis of the relationship between Mϕ2 blood infiltration levels and modules indicated that the turquoise module (R = 0.33, *p* = 1e-04) was the most significantly positively associated with Mϕ2, whereas the blue module (R = −0.37, *p* = 1e-05) was the most significantly negatively associated with Mϕ2 (Figure 4C). Moreover, the turquoise and blue modules were most significantly associated with the clinical diagnosis, which indicates that the two modules might play a critical role in the development from SCAD to AMI and are correlated with Mϕ2. Therefore, the blue and turquoise modules were treated as Mϕ2-related modules for further analyses. Figures 4D,E shows the MEs of the two modules. To ensure the dependability of the identification results for the Mϕ2-related modules, we calculated the mean absolute GS value of the Mϕ2-related genes to identify these modules again. The mean absolute GS values of the blue and turquoise modules were the top two modules with the highest correlations with Mϕ2 (Figure 5A). By applying the two different methods above, we found that the turquoise and blue modules revealed the strongest interconnection with Mϕ2 infiltration. Taken together, we identified the turquoise and blue modules as hub modules for the identification of hub genes.

### Identification of Mϕ2-related MI hub genes

Larger MM values can reflect a higher correlation between genes and Mϕ2. To recognized hub genes in the identified modules, we calculated the MM value for each gene, and then performed a correlation analysis between GS and MM in each module to identify the close connection between the MM value and Mϕ2. The blue and turquoise modules showed the two highest correlation coefficients (blue module = 0.63, *p*-value = 1e-200; turquoise module = −0.47, *p*-value < 1e-200) (Figures 5B,C).

In this study, hub genes were identified by the “network screening” function, which was based on GS and MM. We obtained 482 hub genes that were strongly correlated with Mϕ2, with MM > 0.8 and GS > 0.25 (Supplementary Table 7).

### Functional enrichment analysis of Mϕ2-related hub genes

**FIGURE 6:**
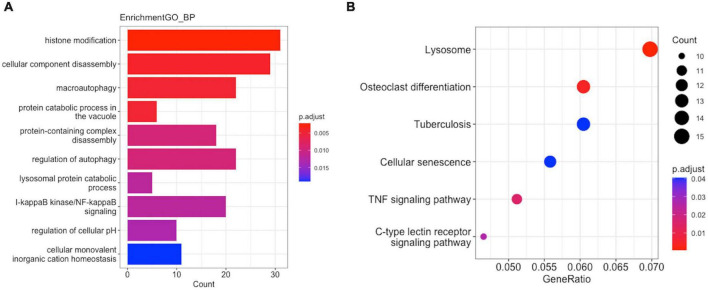
Enrichment analysis of GO function and KEGG pathway in M2-related module hub genes. **(A)** GO analysis. **(B)** KEGG analysis.

To reveal the potential biological functions of the genes in the hub modules, we performed functional enrichment analysis using the clusterProfiler R package (version: 4.1.3). In GO functional enrichment analysis, the Mϕ2-related terms in AMI were the most enriched, including the regulation of histone modification, cellular component disassembly, and macroautophagy (Figure 6A). KEGG analysis revealed that the lysosome, osteoclast differentiation, and tuberculosis pathways were significantly enriched (Figure 6B).

**FIGURE 7:**
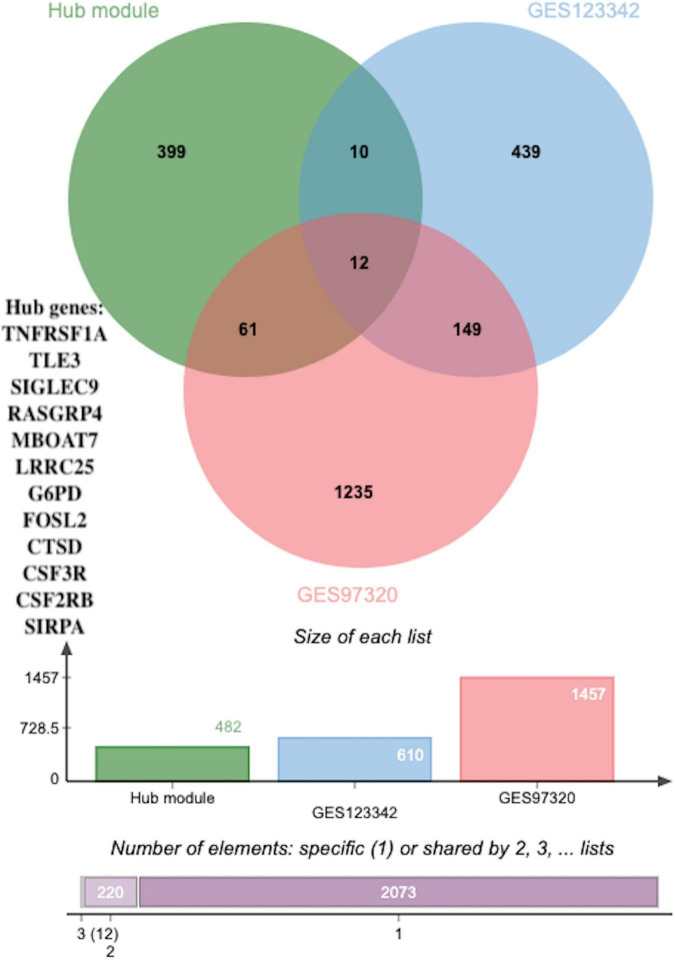
The overlapped hub genes from different databases. Twelve overlapping genes were identified as potential key genes through using a Venn diagram between DEGs (*p* < 0.05 and |log2FC| > 0.5) in GSE123342, DEGs (*p* < 0.05 and |log2FC| > 0.5) in GSE97320 and hub genes.

### Identification and validation of the diagnostic values of the key genes

To identify novel AMI-related diagnostic biomarkers, 631 and 1457 DEGs, with |log2FC| > 0.5 and adjusted *p*-values < 0.05, were screened in GSE123342 and GSE97320, separately (Supplementary Table 8). Subsequent analysis of 631 DEGs in GSE123342, 1457 DEGs in GSE97320, and candidate hub genes identified nine overlapping genes as potential key genes using a Venn diagram (Figure 7).

**FIGURE 8:**
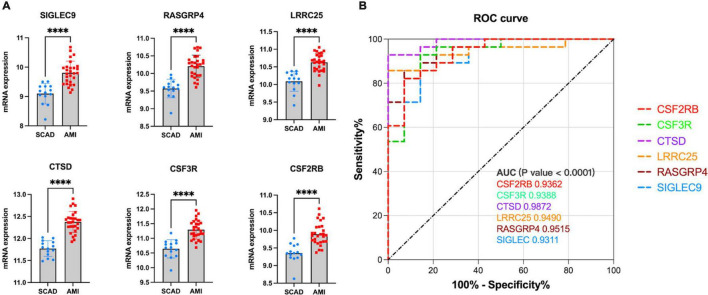
Validation of real hub genes in GSE62646. **(A)** Relative mRNA expression level of six hub genes in SCAD vs. AMI samples. (^****^p < 0.0001). **(B)** ROC curve for six hub genes.

Similarly, to validate their diagnostic and prognostic values and their correlations with clinical features, expression and ROC analyses were performed in GSE62646. Figure 8A shows the differential expression of genes *SIGLEC9* (sialic acid-binding immunoglobulin-like lectin 9), *RASGRP4* (Ras guanine nucleotide-releasing protein-4), *LRRC25* (leucine-rich repeat containing 25), *CTSD* (Cathepsin D), *CSF3R* (colony-stimulating factor-3 receptor), and *CSF2RB* (colony stimulating factor 2 receptor subunit beta) between SCAD and AMI, with *p*-values of <0.0001. All the six key genes were up-regulated in AMI. Furthermore, based on the gene expression levels, ROC curves identified their high diagnostic value as biomarkers for AMI (Figure 8B), with *SIGLEC9, RASGRP4, LRRC25, CTSD, CSF3R*, and *CSF2RB* having AUC values of 0.9,311, 0.9,515, 0.9,490, 0.9,872, 0.9,388, and 0.9,362, respectively.

**FIGURE 9:**
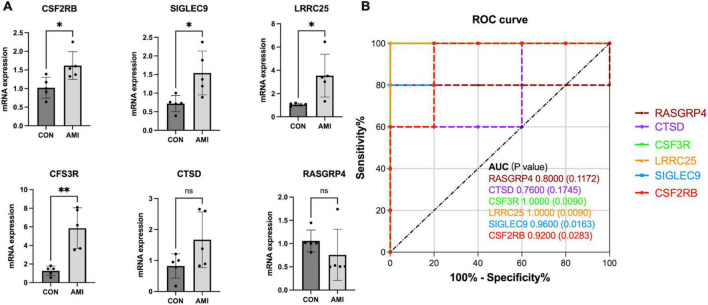
qPCR validation of the expression pattern and evaluation of diagnostic efficacy of six clinical correlated genes in a patient cohort. **(A)** Relative mRNA expression level of six hub genes in healthy controls vs. AMI samples. (*p < 0.05, ^**^p < 0.01). **(B)** ROC curve for six hub genes.

In our cohort, DEGs were only confirmed in four upregulated genes, *SIGLEC9, LRRC25, CSF3R*, and *CSF2RB*, but not in *RASGRP4* and *CTSD* (Figure 9A). Additionally, ROC curves identified high diagnostic value for *SIGLEC9, LRRC25, CSF3R*, and *CSF2RB*, with AUC values of 0.9,600, 1.0.000, 1.0,000, and 0.9,200, separately (Figure 9B). These results indicated that the significantly upregulated genes *SIGLEC9, LRRC25, CSF3R*, and *CSF2RB* could serve as the gene markers to differentiate AMI and controls.

## Discussion

AMI, which is characterized by the sudden obstruction of blood flow to the myocardium, remains a global burden, despite reperfusion strategies and pharmacological treatments having saved many lives (18). The progression of AMI is extremely acute and usually results in delayed treatment periods. Coronary angiography is the primary therapy for the diagnosis and treatment of the disease. Without timely intervention, many people may die. Multiple studies have reported the crucial role of the dys-regulation of distinct immune cells, especially Mϕ, in AMI progression (19–21).

In this study, we constructed distinct modules using WGCNA by selecting 8,760 DEGs and different Mϕ infiltration levels in AMI and SCAD. Highly Mϕ-related gene modules were identified based on correlation coefficients. Hub genes, selected when DEGs had MM values > 0.8 and GS values > 0.25 in GSE59867, were closely related to immune-related genes in GO and KEGG analyses. Twelve overlapping hub genes, identified by Venn diagram analysis of the hub genes and DEGs in GSE123342, and DEGs in GSE97320, were considered potential key genes related to Mϕ2 using GSE62646 as the validation set. Compared with SCAD, only six hub genes (*SIGLEC9, RASGRP4, LRRC25, CTSD, CSF3R*, and *CSF2RB*) had potential diagnostic value and were deemed potential novel prognostic biomarkers for AMI. In comparison with previous studies, our study provides insights into the potential pathogenesis of AMI.

*SIGLEC9*, a cell surface *trans*-membrane receptor, is expressed predominantly on myeloid cells, including monocytes, macrophages, and dendritic cells. By regulating endocytosis of Toll-like receptor 4, *SIGLEC9* participates in macrophage polarization, and inhibits the capacity of neutrophils during infections (22). Aberrant glycosylation during tumor progression is a key hallmark of cancer, resulting in increased sialylation and modulation of the tumor immunological microenvironment (23). *SIGLEC-E* represents the mouse ortholog of human *SIGLEC9* and has been reported to interact with the scavenger receptor CD36, which is involved in modified LDL uptake by suppressing downstream VAV signaling (24). However, the roles of *SIGLEC9* in AMI has not yet been explored.

*CSF3R*, known as the receptor for granulocyte colony–stimulating factor, mainly participates in the regulation of inflammation. *CSF3R/CSF3* are linked to neutrophil development *in vivo*. Several researchers have explored its involvement in the progression of other diseases, including chronic neutrophilic leukemia, asthma, and fatty liver disease (25–27). However, its role in AMI progression remains unknown.

*LRRC25*, a member of the leucine-rich repeat (LRR)-containing protein family, is a *trans*-membrane protein related to autophagy. It acts as a vital negative regulator of the type I interferon (IFN) (28) and nuclear factor kappa-B (NF-κB) (29) signaling pathways, inhibits the generation of inflammatory cytokines, and regulates the response to viral infections. Similar results were shown in *LRRC25*-knockout mice; an *LRRC25* deficiency significantly accelerates pathological cardiac hypertrophy in mice by increasing the NF-κB and TGF-β1 activation signaling pathway to increase inflammation (30). However, *LRRC25* is mainly expressed in monocytes, dendritic cells, granulocytes, and T lymphocytes, and its role in macrophages associated with AMI remains unknown.

*CSF2RB* is also known as a receptor for granulocyte-macrophage colony-stimulating factor. CSF2 is significantly secreted in response to distinct types of injuries, such as AMI, suggesting a significant role in AMI progression (31, 32). As an endogenous cytokine, CSF2 activation of cardiac-resident Ly6C^Lo^ Mϕ is important for the myocardial adaptive response to pressure overload (33). A recent study revealed that irisin upregulates *CSF2RB* expression to induce cardiac homing of adipocyte-derived stem cells, delivered intravenously (34).

*CTSD* (cathepsin D), is a lysosomal aspartic protease, involved in the regulation of lysosomal proteolytic activity. Numerous studies on *CTSD* have shown its importance in the pathogenic mechanism of ischemic heart disease (ICD) (35, 36); the up-regulated expression of *CTSD* in ischemic cardiac muscle accelerates autophagic flux and inhibits cardiac remodeling and further heart failure. The expression of the precursor form of *CTSD* was significantly increased in failing human hearts with ICD.

*RASGRP4*, an activator of the Ras protein, is a receptor associated with the guanine nucleotide exchange factor, diacylglycerol/phorbol ester, and evolutionarily conserved calcium-regulation. *RASGRP4* promotes the renal inflammatory injury mediated by peripheral blood mononuclear cells in diabetes; thus, it plays a significant role in regulating immune activation and the inflammatory response (37). Accumulating evidence on *RASGRP4* has revealed its underlying role in the regulation of leukemia and autoimmune diseases (38–40). However, the role of *RASGRP4* in AMI has not been explored.

Nevertheless, *SIGLEC9, RASGRP4, LRRC25*, and *CSF3R* have not been previously reported to be associated with AMI. In our study, the associations of hub modules with Mϕ0 are in reverse with Mϕ2, which indicates that the module genes may play a part in the macrophage polarization from the unpolarized Mϕ0 to polarized Mϕ2. Based on our results and those of previous studies, we hypothesized that *SIGLEC9, RASGRP4, LRRC25*, and *CSF3R* can influence Mϕ2 polarization of the myocardium in the pathological progression of AMI. However, further research on the characteristics of *SIGLEC9, RASGRP4, LRRC25*, and *CSF3R* is required to verify their correlation with AMI.

Generally, an AUC of 0.5 represents no discrimination; 0.7–0.8 represents acceptable; 0.8–0.9 represents excellent; and >0.9 is considered outstanding (41). All AUCs of *SIGLEC9, RASGRP4, LRRC25, CTSD, CSF3R*, and *CSF2R* were identified as outstanding, indicating the powerful capacity to distinguish between AMI and SCAD. However, in our patient cohort, compared to healthy controls, only four genes (*SIGLEC9, LRRC25, CSF3R*, and *CSF2RB*) were significantly overexpressed with high diagnostic value. A large sample size is required to verify the effectiveness of *SIGLEC9, CSF2RB, LRRC25*, and *CSF3R* as biomarkers for AMI in the future.

## Conclusion

Immune cells infiltration analysis indicated a complex network of regulation in cardiovascular disease, whereas Mϕ plays a significant role in the immune regulation network in myocardial infarction. This is our first attempt to identify novel Mϕ2-related biomarkers in the progression of AMI by applying the CIBERSORT and WGCNA algorithms. *SIGLEC9, LRRC25, CSF3R*, and *CSF2RB*, identified through various validations, were all up-regulated in AMI. Additionally, Mϕ2 was negatively correlated with six potential diagnostic biomarkers. It may be of great significance to study the mechanism between Mϕ and key genes involved in the occurrence and progression of AMI. Our findings provide novel insights into AMI at the Mϕ and molecular levels; however, further *in vivo* or *in vitro* experiments on these key genes are needed to validate their effects on AMI.

## Data availability statement

The original contributions presented in this study are included in the article/Supplementary material, further inquiries can be directed to the corresponding author/s.

## Ethics statement

The studies involving human participants were reviewed and approved by the Medical Ethics Committee of Xiangya Hospital of Central South University. The patients/participants provided their written informed consent to participate in this study.

## Author contributions

RS: conception and design of the study. QZ: data curation, data analysis, and original draft writing. GZ: data acquisition and editing. ZL: interpret the results, experimental validation and revision, and reviewing. JZ: figures preparations and proofread the references. All authors contributed to the article and agreed with the submission.

## Footnotes

1
http://www.ncbi.nlm.nih.gov/geo/

## Abbreviations

AMI: acute myocardial infarction
AUC: area under the curve
CSF3R: colony-stimulating factor-3 receptor
CSF2RB: colony stimulating factor 2 receptor subunit beta
DEG: differentially expressed genes
GEO: Gene Expression Omnibus
GO: Gene Ontology
GS: gene significance
KEGG: Kyoto Encyclopedia of Genes and Genomes
LRRC25: leucine-rich repeat containing 25
ME: module eigengene; analysis
M ϕ: macrophage
ROC: receiver operating characteristic
SCAD: stable coronary artery disease
SIGLEC9: sialic acid-binding immunoglobulin-like lectin 9
WGCNA: weighted gene co-expression network analysis.
