# Supplementary material for: Identification and exploration of novel M2 macrophage-related biomarkers in the development of acute myocardial infarction
Source: Front Cardiovasc Med. 2022 Nov 10;9:974353. doi: 10.3389/fcvm.2022.974353 (PMC9685672; doi:10.3389/fcvm.2022.974353)
Supplement: Supplementary file 1 [file Table_1.DOCX]

Supplementary Material

# Supplementary Table S1. Clinical characteristics of patients

|  | **Healthy controls (n = 5)** | **AMI patients (n = 5)** |
| --- | --- | --- |
| **Age (mean, range)** | **61.0 (56-66)** | **64.6 (51-70)** |
| **Gender (female, %)** | **2 (40%)** | **1 (20%)** |
| **BMI (mean ± SD, kg/m^2^)** | **21.4 ± 2.0** | **24.4 ± 3.4** |
| **Smoking (%)** | **2 (40%)** | **3 (60%)** |
