# Supplementary material for: Identification and exploration of novel M2 macrophage-related biomarkers in the development of acute myocardial infarction
Source: Front Cardiovasc Med. 2022 Nov 10;9:974353. doi: 10.3389/fcvm.2022.974353 (PMC9685672; doi:10.3389/fcvm.2022.974353)
Supplement: Supplementary file 2 [file Table_2.DOCX]

Supplementary Material

**Supplementary Table S2. Primer sequences for real-time PCR**

| **Target name** |  | **Primer** |
| --- | --- | --- |
| CTSD | F | GCAAACTGCTGGACATCGCTTG |
|  | R | GCCATAGTGGATGTCAAACGAGG |
| CSF2RB | F | ATCCTCCTCTCCAACACCTCC |
|  | R | ACCTCCTTCCTCACCTCCCA |
| SIGLEC9 | F | GGGTGCTGGAGCTGCCTT |
|  | R | GTCACTCCTGATGTGGCTTTGC |
| LRRC25 | F | CTCCACTCCCGACTATGAGAAC |
|  | R | GTTACAGTAGACAGGCTGGGAAG |
| RASGRP4 | F | AGCATGAACAGAAAAGACAGTAAG |
|  | R | TGTCTAGGAATCCGGCTTGGA |
| CSF3R | F | CAGGGCTATGTGATTGAG |
|  | R | TGGTGTAGTGGGTAAGGG |
| GAPDH | F | GTCTCCTCTGACTTCAACAGCG |
|  | R | ACCACCCTGTTGCTGTAGCCAA |
